# Supplementary material for: Pharmacological Inhibition of miR-130 Family Suppresses Bladder Tumor Growth by Targeting Various Oncogenic Pathways via PTPN1
Source: Int J Mol Sci. 2021 Apr 29;22(9):4751. doi: 10.3390/ijms22094751 (PMC8124864; doi:10.3390/ijms22094751)
Supplement: Supplementary file 1 [file ijms-22-04751-s001.zip › ijms-1108861-supplementary.pdf]

Supplementary information

Supplementary tables

Supplementary table 1

Term	Overlap	P-value	Adjusted P-value	Odds Ratio	Combined Score	Proteins
Thymic Stromal LymphoPoietin (TSLP) Signaling Pathway WP2203	6/47	2.E-09	3.E-07	64.74	1296.42	LYN;YES1;SRC;LCK;FYN;MAPK14
NRF2-ARE regulation WP4357	4/21	3.E-07	3.E-05	89.27	1334.97	YES1;SRC;INSR;FYN
EGF/EGFR Signaling Pathway WP437	6/162	3.E-06	2.E-04	16.92	212.87	SRC;PRKCD;ABI1;INPPL1;MAPK14;BCAR1
B Cell Receptor Signaling Pathway WP23	5/97	5.E-06	2.E-04	23.46	287.51	LYN;LCK;PRKCD;FYN;MAPK14
Kit receptor signaling pathway WP304	4/59	2.E-05	4.E-04	30.78	341.14	LYN;SRC;FYN;MAPK14
Genotoxicity pathway WP4286	4/63	2.E-05	4.E-04	28.69	310.45	HIST1H2BN;HIST1H2BM;HIST1H2BB;HIST1H2BC
Oncostatin M Signaling Pathway WP2374	4/65	2.E-05	4.E-04	27.75	296.79	SRC;PRKCD;CDK2;MAPK14
AGE/RAGE pathway WP2324	4/66	2.E-05	4.E-04	27.30	290.33	SRC;INSR;PRKCD;MAPK14
Human Thyroid Stimulating Hormone (TSH) signaling pathway WP2032	4/66	2.E-05	4.E-04	27.30	290.33	SRC;CDK2;MAPK14;IGF1R
Insulin Signaling WP481	5/160	5.E-05	8.E-04	13.88	136.47	INSR;PRKCD;INPPL1;MAPK14;IGF1R
Signaling Pathways in Glioblastoma WP2261	4/82	6.E-05	8.E-04	21.68	212.02	SRC;PRKCD;CDK2;IGF1R
Alpha 6 Beta 4 signaling pathway WP244	3/33	8.E-05	1.E-03	41.50	391.09	SRC;PRKCD;MAPK14
T-Cell antigen Receptor (TCR) Signaling Pathway WP69	4/90	8.E-05	1.E-03	19.66	185.06	LCK;PRKCD;FYN;MAPK14
Pathways Regulating Hippo Signaling WP4540	4/98	1.E-04	1.E-03	17.98	163.29	INSR;PRKCD;IGF1R;EPHA2
Focal Adhesion WP306	5/198	1.E-04	2.E-03	11.13	98.21	SRC;FYN;BCAR1;IGF1R;PPP1CA
Interleukin-11 Signaling Pathway WP2332	3/44	2.E-04	2.E-03	30.35	259.69	YES1;SRC;FYN
Hippo-Merlin Signaling Dysregulation WP4541	4/120	2.E-04	2.E-03	14.55	120.82	INSR;IGF1R;EPHA2;PPP1CA
IL-3 Signaling Pathway WP286	3/49	3.E-04	2.E-03	27.04	222.72	LYN;SRC;FYN
VEGFA-VEGFR2 Signaling Pathway WP3888	5/236	3.E-04	3.E-03	9.28	74.35	SRC;PRKCD;FYN;MAPK14;BCAR1
Interferon type I signaling pathways WP585	3/54	4.E-04	3.E-03	24.38	193.80	LCK;FYN;MAPK14
TGF-beta Signaling Pathway WP366	4/132	4.E-04	3.E-03	13.18	104.64	SRC;CDK1;MAPK14;BCAR1
RANKL/RANK (Receptor activator of NFKB (ligand)) Signaling Pathway WP2018	3/55	4.E-04	3.E-03	23.91	188.77	LYN;SRC;MAPK14
Brain-Derived Neurotrophic Factor (BDNF) signaling pathway WP2380	4/144	5.E-04	4.E-03	12.04	91.64	SRC;PRKCD;FYN;MAPK14
Prolactin Signaling Pathway WP2037	3/76	1.E-03	6.E-03	17.02	118.19	SRC;FYN;MAPK14
Leptin signaling pathway WP2034	3/76	1.E-03	6.E-03	17.02	118.19	SRC;FYN;MAPK14
Type II diabetes mellitus WP1584	2/22	1.E-03	9.E-03	40.67	266.53	INSR;PRKCD
Angiogenesis WP1539	2/24	2.E-03	1.E-02	36.97	235.82	SRC;MAPK14
Follicle Stimulating Hormone (FSH) signaling pathway WP2035	2/27	2.E-03	1.E-02	32.53	199.83	SRC;MAPK14
Integrin-mediated Cell Adhesion WP185	3/101	2.E-03	1.E-02	12.66	77.57	SRC;FYN;BCAR1
Nanoparticle-mediated activation of receptor signaling WP2643	2/28	2.E-03	1.E-02	31.28	189.87	SRC;MAPK14
Senescence and Autophagy in Cancer WP615	3/105	2.E-03	1.E-02	12.16	73.17	SRC;MAPK14;IGF1R
T-Cell Receptor and Co-stimulatory Signaling WP2583	2/29	2.E-03	1.E-02	30.12	180.72	LCK;FYN
Ebola Virus Pathway on Host WP4217	3/129	4.E-03	2.E-02	9.83	53.45	TYRO3;MERTK;IGF1R
ATM Signaling Pathway WP2516	2/40	5.E-03	2.E-02	21.39	114.76	CDK2;CDK1
IL-2 Signaling Pathway WP49	2/42	5.E-03	2.E-02	20.32	107.07	LCK;FYN
Metabolic reprogramming in colon cancer WP4290	2/42	5.E-03	2.E-02	20.32	107.07	LDHA;GART
Integrated Cancer Pathway WP1971	2/44	6.E-03	2.E-02	19.35	100.20	CDK2;CDK1
Aryl Hydrocarbon Receptor WP2586	2/46	6.E-03	3.E-02	18.46	94.03	SRC;CDK2
Regulation of Microtubule Cytoskeleton WP2038	2/46	6.E-03	3.E-02	18.46	94.03	SRC;CDK1
Chemokine signaling pathway WP3929	3/164	8.E-03	3.E-02	7.68	36.67	LYN;PRKCD;BCAR1
RIG-I-like Receptor Signaling WP3865	2/60	1.E-02	4.E-02	14.00	64.09	DDX17;MAPK14
Notch Signaling Pathway WP61	2/61	1.E-02	4.E-02	13.76	62.57	LCK;SRC
T-Cell antigen Receptor (TCR) pathway during Staphylococcus aureus infection WP3863	2/62	1.E-02	4.E-02	13.53	61.10	LCK;FYN
PI3K-Akt Signaling Pathway WP4172	4/340	1.E-02	4.E-02	4.97	22.43	INSR;CDK2;IGF1R;EPHA2
Ras Signaling WP4223	3/184	1.E-02	4.E-02	6.83	30.46	INSR;IGF1R;EPHA2
G1 to S cell cycle control WP45	2/64	1.E-02	4.E-02	13.09	58.33	CDK2;CDK1
Folate Metabolism WP176	2/66	1.E-02	4.E-02	12.68	55.75	INSR;GART
DNA Damage Response WP707	2/68	1.E-02	4.E-02	12.30	53.36	CDK2;CDK1
RAC1/PAK1/p38/MMP2 Pathway WP3303	2/68	1.E-02	4.E-02	12.30	53.36	SRC;MAPK14
Non-genomic actions of 1,25 dihydroxyvitamin D3 WP4341	2/71	1.E-02	5.E-02	11.76	50.06	PRKCD;MAPK14
miRNA Regulation of DNA Damage Response WP1530	2/71	1.E-02	5.E-02	11.76	50.06	CDK2;CDK1
Primary Focal Segmental Glomerulosclerosis FSGS WP2572	2/72	1.E-02	5.E-02	11.59	49.04	FYN;MKI67
Robo4 and VEGF Signaling Pathways Crosstalk WP3943	1/6	2.E-02	5.E-02	79.78	333.96	SRC
Somatroph axis (GH) and its relationship to dietary restriction and aging WP4186	1/6	2.E-02	5.E-02	79.78	333.96	IGF1R

Supplementary table 2

Protein name	# Peptides	# Unique peptides	# protein hitsMS/MS Count	Posterior error probability	Score	Ratio H/L variability
**HMGN4**	4	3	3	1.2458E-17, 5.80E-71	148.44, 273.82	14.283
**ARPC2**	6	6	6	4.E-03, 3.E-04, 2.E-04	82.287, 82.489, 89.913	26.36
**HNRNPR**	17	10	8	1.E-02, 4.E-11, 4.E-11, 3.E-03, 5.E-03, 2.E-05	45.764, 105.39, 96.123, 62.617, 49.298, 80.312	17.904
**BUB3**	5	5	6	3.E-08, 2.E-28, 2.E-08	80.412, 196.37, 86.959	10.758
**SRPRB**	6	6	5	9.E-21, 4.E-23, 3.E-12	138.19, 151.95, 105.79	30.219
**RPL24**	16	16	9	1.E-02, 1.E-05, 1.E-05, 6.E-09, 3.E-10	62.287, 102.4, 68.168, 117.94, 109.99	26.775
**SRSF3**	12	12	17	2.E-03, 1.E-05, 6.E-04, 2.E-10, 3.E-02, 5.E-02, 6.E-12	76.165, 98.105, 67.11, 82.665, 44.543, 32.518, 162.93	17.974
**SRSF2**	5	5	8	1.E-03, 8.E-02, 1.E-04, 5.E-05	85.807, 61.325, 84.479, 92.781	14.526
**CAP1**	11	11	5	2.E-09, 2.E-09, 2.E-03, 2.E-03, 8.E-34	131.35, 124.07, 90.601, 69.846, 192.62	21.029
**SFXN1**	11	11	10	2.E-02, 9.E-03, 8.E-05	55.776, 84.097, 75.316	17.22
**WDR1**	23	23	10	5.E-93, 3.E-06, 5.E-02, 2.E-10, 4.E-14, 4.E-105, 7.E-02	246.55, 79.346, 39.645, 130.2, 98.3, 240.07, 64.841	15.041
**HIST1H1E**	17	3	51	1.E-02, 1.E-12, 5.E-08, 7.E-56, 8.E-08, 3.E-07, 4.E-06, 8.E-04, 2.E-04, 3.E-28, 8.E-37, 1.E-06	54.898, 112.58, 101.79, 256.93, 87.352, 116.13, 86.498,	12.611
**HSPA8**	38	33	92	2.E-09, 5.E-34, 2.E-07, 5.E-06, 8.E-03, 6.E-10, 9.E-21, 7.E-42, 4.E-02, 8.E-20, 7.E-20, 6.E-03, 2.E-20, 4.E-26, 2.E-16, 3.E-77, 1.E-59, 8.E-57, 1.E-107, 3.E-12, 5.E-33, 1.E-10, 4.E-24	124.62, 181.49, 100.93, 86.803, 86.332, 120.9, 138.66, 185.41, 45.347, 127.92, 118.37, 68.107, 171.76, 185.67 110.29, 281.99, 217.71, 183.93, 314.57, 156.08, 177.09 79.949, 133.36	19.365
**PTPN1**	4	4	6	9.E-06, 4.E-03	78.615, 56.139	34.063
**TMOD3**	5	5	4	9.E-10, 5.E-02, 1.E-17	131.36, 48.848, 128.74	23.715
**SLC7A5**	3	3	7	2.E-10, 8.E-17	109.79, 135.08	17.153
**TWF1**	8	8	7	2.E-05, 3.E-02, 4.E-03, 3.E-33	89.663, 77.282, 74.29, 181.64	17.543
**HNRNPA0**	8	8	15	1.E-07, 9.E-04, 1.E-16, 1.E-14	91.065, 61.265, 126.04, 136.57	18.961
**EIF3I**	15	15	16	2.E-03, 2.E-02, 4.E-04, 8.E-06, 1.E-39, 2.E-05	71.781, 81.92, 75.088, 146.11, 234.85, 79.492	23.302
**SHPK**	6	6	4	2.E-04, 3.E-04, 4.E-07	59.372, 68.044, 97.273	27.929
**VCL**	44	44	31	1.E-19, 5.E-15, 1.E-03, 1.E-23, 3.E-17, 2.E-11, 7.E-15, 3.E-02, 3.E-08, 8.E-03, 3.E-03, 4.E-26, 4.E-06, 8.E-10 3.E-14, 6.E-02, 4.E-03, 6.E-03, 4.E-03, 5.E-54	127.5, 134.14, 77.597, 154.26, 120.71, 152.32, 119.87 54.823, 121.18, 58.172, 67.214, 181.69, 109.44, 100.11 128.59, 25.408, 104.42, 52.821, 115.49, 245.96	14.42
**MAP4**	22	22	30	8.E-20, 5.E-03, 9.E-06, 9.E-02, 2.E-08, 7.E-13, 1.E-15 8.E-02, 2.E-04, 5.E-04, 3.E-03, 5.E-10, 2.E-09, 2.E-20 1.E-05, 3.E-04	142.85, 68.856, 89.507, 24.229, 153.34, 156.17, 113.65 42.719, 68.283, 54.869, 49.721, 100.69, 101.42, 144.08 69.256, 62.69	22.567
**MAPK1**	6	5	2	7.00E-06	112.84	14.533
**RPL13A**	6	6	8	2.E-02, 1.E-05, 3.E-04	75.189, 107.21, 82.445	28.572
**CDV3**	7	7	10	2.E-186, 2.E-09, 8.E-34, 1.E-27, 1.E-03, 4.E-94, 6.E-05	321.96, 110.78, 203.27, 169.66, 85.676, 231.65, 67.396	20.744
**PTGES3**	7	7	5	2.E-07, 6.E-03, 5.E-02, 3.E-04	115.65, 47.712, 71.141, 80.014	24.273
**MAPRE1**	7	7	9	1.E-26, 6.E-41, 3.E-08	143.52, 202.6, 81.917	22.254
**TIMM50**	12	12	15	2.E-33, 3.E-11, 1.E-08, 4.E-07, 3.E-17	188.27, 107.03, 127.27, 108.97, 133.94	25.756
**PABPN1**	7	7	21	2.E-14, 1.E-41, 2.E-15, 3.E-36	127.71, 179.46, 115.39, 154.29	20.724
**MTPN**	5	5	3	3.E-15, 1.E-37	107.75, 199.61	13.339
**LRPPRC**	7	7	4	8.E-09, 5.E-02	124.62, 32.859	21.259
**RPL5**	16	16	29	3.E-03, 1.E-05, 2.E-02, 2.E-02, 6.E-11, 7.E-02, 3.E-51, 1.E-04, 2.E-12	74.841, 82.651, 100.74, 48.284, 138.21, 41.164, 184.3 93.096, 148.52	25.779
**METAP1**	5	5	11	3.E-28, 3.E-06, 1.E-44, 7.E-15	159.98, 90.793, 195.18, 115.39	22.265
**ADK**	6	6	5	2.E-05, 2.E-02, 4.E-06, 8.E-06	101.38, 40.883, 79.639, 80.17	17.903
**ABCE1**	8	8	3	8.E-02, 1.E-10	21.886, 140.91	23.293
**SERBP1**	8	8	3	6.E-24, 4.E-03	125.65, 47.855	25.112
**FERMT2**	4	4	3	6.E-24, 4.E-03	125.65, 47.855	26.37
**FUBP1**	14	9	17	4.E-07, 9.E-06, 4.E-02, 2.E-21, 2.E-02, 2.E-41	78.708, 143.61, 45.28, 194.65, 47.774, 177.08	17.083
**ERP44**	3	3	4	8.E-05	103.26	14.509
**RPS15A**	4	4	7	6.E-04, 1.E-06, 9.E-02, 7.E-44	84.507, 97.463, 7.8731, 194.19	16.459
**RPL8**	25	25	46	1.E-08, 8.E-26, 4.E-11, 1.E-33, 3.E-03, 1.E-04, 2.E-06, 1.E-04, 5.E-04, 2.E-10, 2.E-02, 9.E-03, 5.E-03, 3.E-03 1.E-02, 1.E-02	93.505, 176.44, 104.88, 190.23, 66.056, 83.54, 91.62, 74.987, 147.08, 81.315, 76.17, 48.585, 98.009, 87.639, 97.596, 81.784	22.179

Supplementary Figures

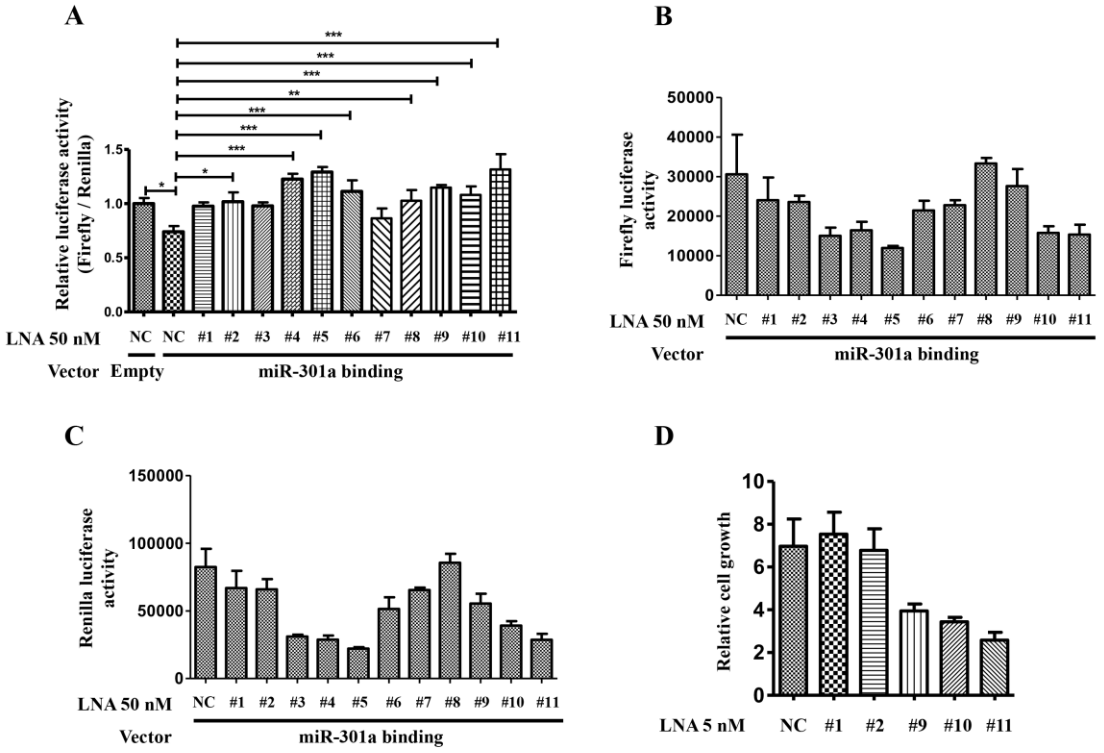

Supplementary Figure 1. Screening of 10 candidate miR-130 family-targeted LNAs.

A dual-luciferase reporter assay was performed using 5637 cells transfected with miR-130 family-targeted LNAs. Data were analyzed using one-way ANOVA with Bonferroni post-hoc test *p < 0.05; **p < 0.01. Data are presented as the mean ± SD (n = 3). The results are shown as firefly luciferase activity relative to (Renilla luciferase activity (A), firefly luciferase activity (B), and Renilla luciferase activity (C). (D) The effect of candidate miR-130 family-targeted LNAs on 5637 cell growth was determined using WST-8 reagent. Data are presented as the mean ± SD. (n = 4). Data were analyzed using one-way ANOVA with Bonferroni post-hoc test **p < 0.01; ***p < 0.001.

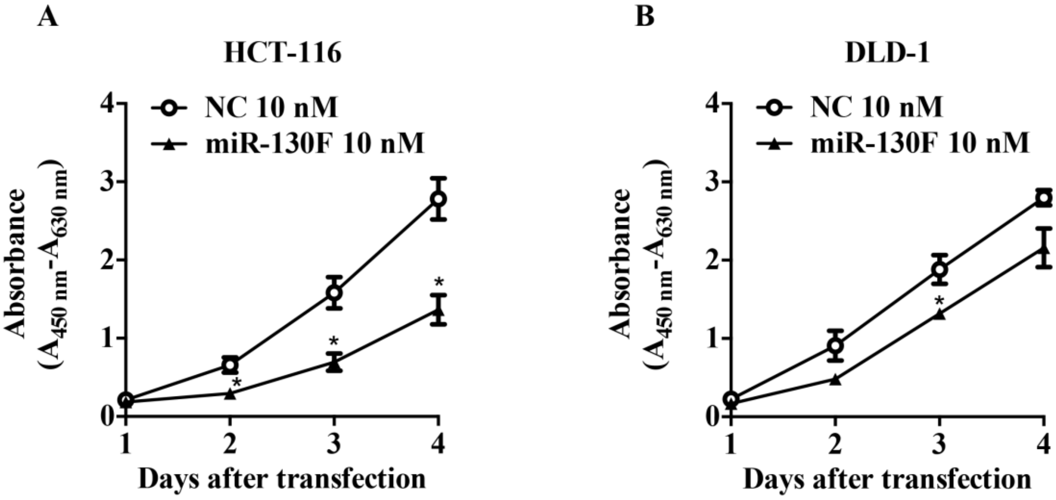

Supplementary Figure 2. The effect of miR-130 family-targeted LNA (miR-130F) on the proliferation of colon cancer cell lines.

The effect of miR-130 family-targeted LNA (miR-130F) on the proliferation of HCT-116 (A) or DLD-1 (B) cells was measured using a WST-8 assay. Data are presented as the mean ± SD (n = 3). Data were analyzed using Unpaired t test *$p < 0.05$.

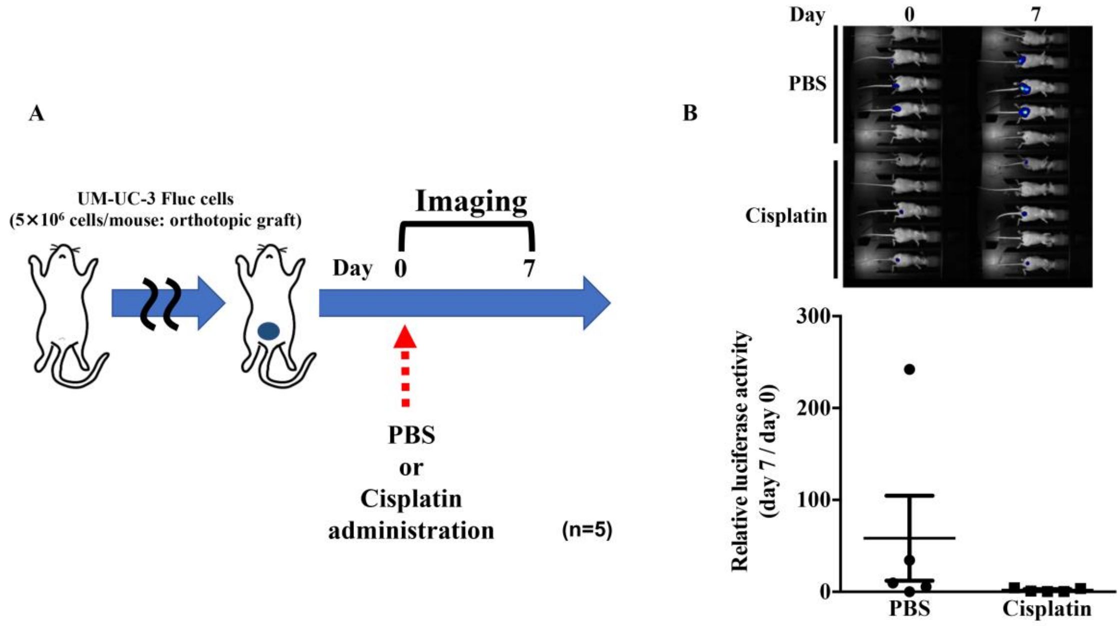

Supplementary Figure 3. Verification of an orthotopic bladder cancer model using cisplatin. (A) Scheme of miR-130 family-targeted LNA challenge in an orthotopic bladder cancer model. PBS or cisplatin was administered in UM-UC-3 cell-inoculated mice at a dose of 10 mg/kg. (B) Relative tumor volume was measured by *in vivo* imaging. Data are presented as the mean ± SD. (n = 5).

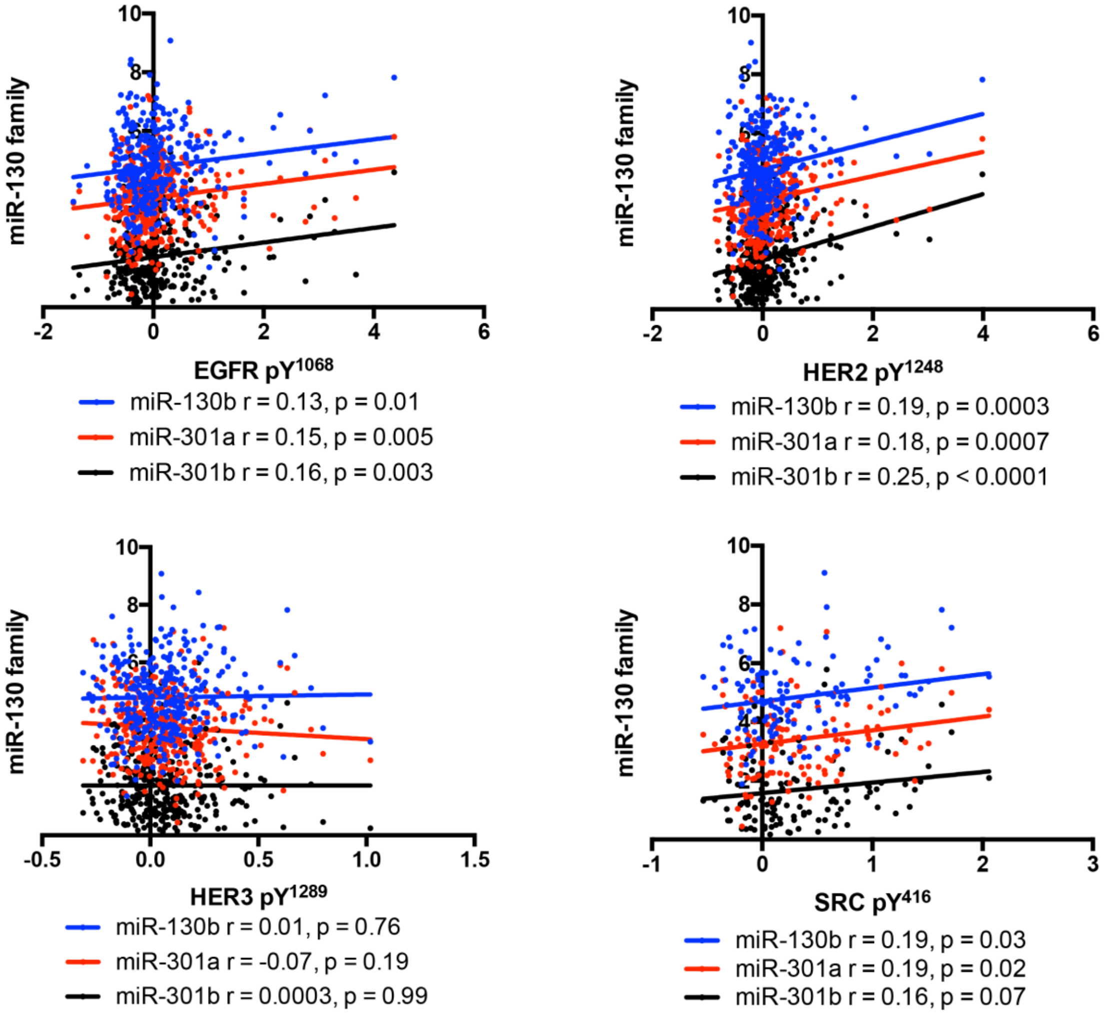

Supplementary Figure 4. Correlation analysis of the expressions between miR-130 family and phosphorylated receptor tyrosine kinases in bladder cancer specimens. Correlation analysis was conducted using University of California, Santa Cruz (UCSC) Xena database (http://xena.ucsc.edu).

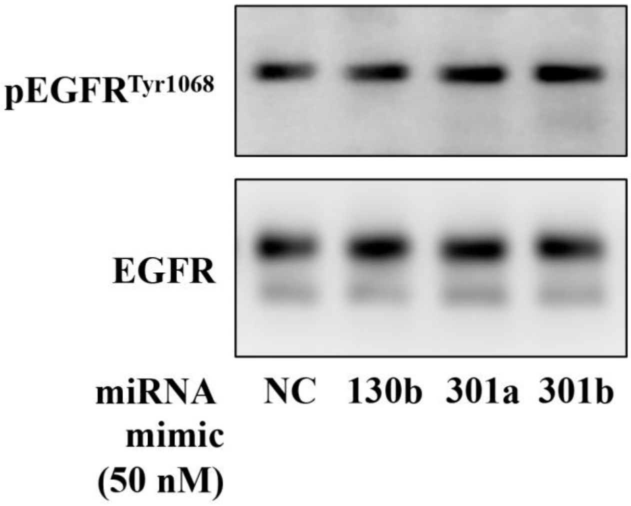

Supplementary Figure 5. The effect of miR-130 family mimics on EGFR phosphorylation levels.

miR-130 family mimics were transfected into UM-UC-2 cells. Protein expression levels of pEGFR^{Tyr1068} were evaluated by western blot analysis. Representative pictures of three independent experiments are shown. Uncropped western blot data are shown in Supplementary Fig. 6.

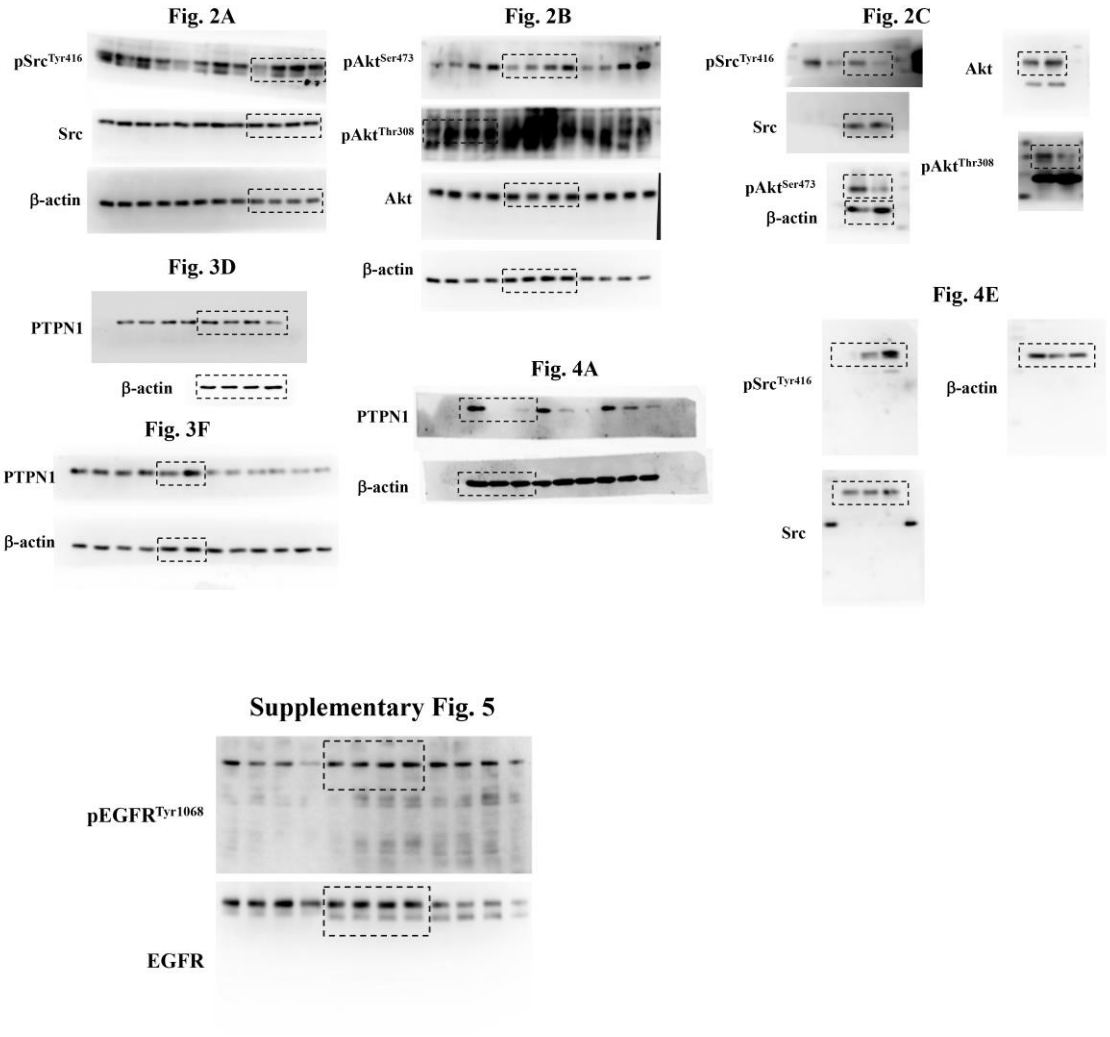

Supplementary Figure 6. Uncropped western blot data.
